# When Psychiatric Services Become a Waiting Room: Situational Analysis of Involuntary Commitment and Treatment as Experienced by Patients and Nurses

**DOI:** 10.1177/10547738251321067

**Published:** 2025-03-12

**Authors:** Pierre Pariseau-Legault, David Pelosse, Emmanuelle Bernheim, Marie-Hélène Goulet, Guillaume Ouellet, Lisandre Labrecque-Lebeau, Jean-Daniel Jacob, Dave Holmes

**Affiliations:** 1 Université du Québec en Outaouais, Saint-Jerome, QC, Canada; 2 University of Ottawa, Ottawa, ON, Canada; 3 Université de Montréal, Montréal, QC, Canada; 4 Centre de recherche de Montréal sur les inégalités sociales, les discriminations et les pratiques alternatives de citoyenneté (CREMIS), Montreal, QC, Canada

**Keywords:** psychiatry, mental health, coercion, involuntary commitment, involuntary treatment, community treatment orders

## Abstract

A growing body of literature highlights the involvement of nurses in the application of involuntary commitment and treatments in psychiatry. The violence underlying these coercive practices is often discussed, as they infringe on human rights and have negative effects on both patients and healthcare staff. The current state of knowledge on this subject, however, fails to inform us of what characterizes and influences these practices in psychiatric nursing. A situational analysis was conducted to gain a better understanding of this issue. This qualitative research aims to explore the characteristics of nursing care during involuntary commitment and treatments. In all, 10 nurses (*n* = 10) and 11 patients (*n* = 11) participated in semi-structured interviews and completed a sociodemographic questionnaire. Data analysis followed a grounded theory approach, involving a process of coding, conceptualizing, categorizing, constant comparison, and relational mapping, accompanied by analytical memos. Four conceptual categories emerged from data analysis: (1) Psychiatry as a waiting room, (2) nurses as subordinates, (3) nothing else but medication, and (4) resisting undignifying care. The results suggest that clinical issues surrounding involuntary commitment and treatments can be explained by how care is conceived. The psychiatric nursing practice seems to be limited to the application of coercive power, such as forced administration of medication. The distress potentially induced by involuntary commitment and treatments in patients comes to be ignored in favor of compliance with the legal procedures. The results describe a situation where patients felt abandoned to those procedures as if refusing to be hospitalized or treated were incompatible with any other form of care. Several participants also report having suffered negative consequences following one or more coerced psychiatric episodes. For them, refusal of care therefore seems to be associated with a resistance against the current violence of biomedical psychiatry, rather than a refusal to obtain help and support.

## Introduction

A growing body of literature highlights the involvement of nurses in the application of involuntary commitment and treatments in psychiatry (Haines et al., 2024; Lessard-Deschênes & Goulet, 2022; Manderius et al., 2023). Yet, very little is known about psychiatric nursing practice in this context. Studies on involuntary commitment (also known as forced hospitalization or involuntary admission) reveal predominantly negative experiences among patients and healthcare professionals. These experiences are marked by a significant power imbalance, often manifesting as a lack of information sharing with patients (Aragonés-Calleja & Sánchez-Martínez, 2024). The effectiveness of involuntary treatments (also known as court-ordered treatments, involuntary treatment orders, or community treatment orders) has not been demonstrated when compared to treatments provided voluntarily. Moreover, the harms of such measures on patients remain understudied (Haines et al., 2024; Kisely et al., 2024), and many argue that psychiatric coercion cannot be justified ethically, legally, or clinically (Hempeler et al., 2024; Martin & Gurbai, 2019; Richter, 2024).

The use of involuntary commitment and treatments is also at odds with the principles of recovery-oriented and trauma-informed care (Crowe, 2022; Haines et al., 2024; Torrents & Björkdahl, 2024), which prioritize shared decision-making, trusting relationships, and the preservation of patients’ dignity. Some authors argue that recovery-oriented and trauma-informed care is still achievable despite psychiatric coercion, provided they consider the relational aspects of care, patient agency, information-sharing challenges, and environment safety (McKay et al., 2021; Prytherch et al., 2021). Psychiatric advance directives are also proposed as an alternative strategy to psychiatric coercion, aiming to better address the ethical challenges posed by involuntary commitment and treatments (Hempeler et al., 2024; Torrents & Björkdahl, 2024). There is a growing trend advocating for the replacement of psychiatric coercion with preventive and person-centered approaches, focused on agency, collaboration, and human rights (Evans et al., 2024; Sugiura et al., 2019; Watson et al., 2014). However, a recent study on the abolition of psychiatric coercion indicates that only a minority of participants consider this goal realistic, given the complexities of mental health treatment and safety concerns (Birkeland et al., 2024).

Consequently, and perhaps paradoxically, studies show these coercive practices are rising globally (Lebenbaum et al., 2018; Sashidharan et al., 2019), still being an important part of psychiatry (Korezelidou et al., 2025). Despite their negative effects on both patients and healthcare staff (Aragonés-Calleja & Sánchez-Martínez, 2024; Corderoy et al., 2024; Mooney & Kanyeredzi, 2021), coercive practices are now integrated into the daily work of nurses (Wand, 2024), considered a “necessary evil” (Doedens et al., 2020, p. 450) and thus normalized (Markham 2024). Jenkins et al. (2023) add that nurses, despite their benevolent intentions, become agents of control by enforcing harmful laws on patients. These findings are supported by Lipsky (2010), who describes the role of state agents as an important part of the street-level bureaucracy. These agents are endowed with significant discretionary power in the implementation of public policies (Chang & Brewer, 2023). The implementation of deleterious laws may lead patients to reject the biomedical model that currently organizes psychiatric services, which is itself deeply intertwined with coercive practices (Johansson et al., 2024; Kirk, 2017; Sørås & Snipstad, 2021; Szasz, 2009). As a result, some patients are in a situation of non-take-up of services intended for them (Warin, 2016), due to prior negative experiences with these services, the lack of alternatives to medication, and fear of being subjected to involuntary commitment or treatments in the event of refusal (Johansson et al., 2024; Prytherch et al., 2021).

Emerson and Pollner (1976, p. 243) have also raised troubling observations about psychiatric care practices, which tend to be perceived as “dirty work.” They emphasize that some clinicians, particularly nurses, feel incapable of acting therapeutically during involuntary commitment. The designation of dirty work allows professionals to view these coercive interventions as an exception to their therapeutic role, thereby preserving their integrity. More than 50 years have passed since Emerson and Pollner’s (1976) conclusions. And yet, Godin (2000) and McKeown (2024) support the relevance of this observation today by describing nursing work in psychiatry as a “bullshit job,” partly due to the increasingly coercive role of nurses. Considering that the use of involuntary commitment and treatments has become routine for many nurses (Wand, 2024), with numerous negative consequences on patients, this situation underscores the urgency for reflection on the evolution of psychiatric care practices, particularly in coercive contexts.

## Context

In Quebec (Canada), involuntary commitment and treatments are two distinct legal measures. Involuntary treatment, also known as community treatment orders, allows for the imposition of care in various situations, including when an adult is deemed incapable of giving consent and refuses treatment if such treatment is required by his or her condition. It is initiated by a request from a healthcare institution or a physician to the court, authorizing healthcare personnel to act against the person’s will (Government of Québec, 2024a). By contrast, involuntary commitment permits the admission of persons against their will if they pose a danger to themselves or others, without imposing treatment. An initial 72-hr period of hospitalization may be imposed by authorized professionals without a court order. If hospitalization extends beyond the legally authorized period, a court order must be obtained. A psychiatric evaluation is conducted with the person’s consent or is also ordered by the court in case of refusal. At the very beginning of involuntary commitment, healthcare personnel must inform the person of the reasons for their admission, their location, and their right to communicate with relatives or a lawyer. In addition, a document outlining the rights of patients must be provided to them following the court order extending the period of hospitalization. Recently, nurse practitioners have been included with physicians under this law, allowing them to impose involuntary commitment for a maximum of 72 hr, terminate the hospitalization, and restrict certain communications, under specific conditions (Government of Québec, 2024b).

Many countries, including England, Australia, and New Zealand (National Archives, 1983; Royal Australian and New Zealand College of Psychiatrists, 2017), have laws permitting the imposition of care during involuntary commitment, as well as involuntary commitment in cases of treatment non-compliance. Involuntary commitment and treatment are based on a fundamental critical process: the temporary suspension of freedom to make autonomous decisions regarding standard psychiatric care, as stipulated by law. In this study, we included participants with experiences of involuntary commitment and treatments without differentiation, considering that they both represent types of care involving nurses and that participants were likely to have experienced these two measures. By doing so, we aimed to better understand the general characteristics of such care, particularly the support provided to individuals undergoing these experiences.

## Aims and Objectives

This research aims to gain a better understanding of involuntary commitment and treatments as they manifest in psychiatric nursing. We aimed to address the following research question: How is psychiatric care perceived by nurses and patients experiencing involuntary commitment and treatments? The objectives of this study are to (1) identify the characteristics of nursing care from the perspective of patients and nurses and (2) describe the factors influencing the practices deployed by psychiatric nurses during involuntary commitment and treatments.

## Methodology

An exploratory qualitative research design was employed to address the research aim, question and objectives. A situational analysis was conducted (Clarke et al., 2017), which is a methodological approach emerging from the interpretive turn in grounded theory. Situational analysis is often described as deriving from grounded theory. However, its purpose is to provide a detailed description of a social phenomenon (the situation) by situating it within its broader context (Clarke et al., 2022), rather than developing a theory. The situation itself is the central focus of the analysis, as opposed to human action and social process, which is the focal point in grounded theory (Clarke et al., 2022). Situational analysis places particular emphasis on contextual sensitivity, power relations, and the use of a variety of discourses and empirical material to provide a rich and detailed description of the analyzed situation. A distinctive feature of this approach is the use of different mapping strategies to relate and analyze all human and non-human elements within a given situation. These maps are of three types: situational, positional, and social arenas (Clarke et al., 2017). They are available for open-access consultation (Pariseau-Legault, 2024).

### Data Collection

Participant recruitment was carried out through purposive sampling via social media and community mental health advocacy organizations. Between March 2021 and December 2023, 21 participants (*n* = 21) were involved, including 10 nurses who participated in involuntary commitment or treatments in the last 5 years, and 11 patients who have experienced involuntary commitment or treatments in the last 10 years. Each participant completed a semi-structured interview lasting approximately 90 min and completed a sociodemographic questionnaire. The interview guides were developed based on existing literature on the subject (Pariseau-Legault et al., 2020). They were further adjusted following non-participant observations (*n* = 70 hr) and a review of case law (*n* = 126 judgments) to provide a deeper understanding of the challenges associated with nursing practices during involuntary commitment and treatments. The observations were carried out with mental health advocacy organizations and during public hearings of the Quebec Coroner’s Office regarding deaths by suicide. The case law review was carried out using the Canadian Legal Information Institute database (CanLII). Interview guides are available for open-access consultation (Pariseau-Legault, 2024).

### Data Analysis

Data collection, transcription, and analysis were conducted iteratively, typically grouping three to four interviews with parallel observations. Interviews and observation notes were manually transcribed. All data were managed using NVivo© software. According to the principles of situational analysis, several sensitizing concepts emerged, forming the framework for this research: dirty work (Emerson & Pollner, 1976; Godin, 2000; McKeown, 2024), street-level bureaucracy (Lipsky, 2010), and non-take-up of social policies (Warin, 2016). These concepts guided the interpretation of the results and are integrated into the discussion. Theoretical saturation, defined as the point when no new elements need to be integrated into the situational maps (Clarke et al., 2017), was reached after 9 interviews with nurses and 10 with patients. An additional interview per participant category was conducted to confirm this saturation. Data analysis followed a grounded theory approach, involving a process of coding, conceptualizing, categorizing, constant comparison, and relational mapping, accompanied by analytical memos (Clarke et al., 2017; Corbin & Strauss, 1990).

### Rigor and Reflexivity

Various strategies were employed to ensure the credibility, reliability, transferability, and internal consistency of the results (Gohier, 2004). Data analysis was carried out by consensus between PPL and GO (nurses) and PPL and DP (patients), with the participation of all authors during the final analysis. PPL kept a research log to document the research and analysis process. Data were triangulated with case law review and non-participant observations. Situational analysis also involves clarifying the researchers’ positionality. As principal investigator, PPL is a psychiatric nurse who has been involved in coercive measures, which may have influenced the interpretation of the data.

## Results

The sociodemographic data of the participants are presented in Tables 1 and 2. For comparison purposes, we also used close-ended questions to ask both nurses and patients to share their general perceptions regarding various dimensions of coercion (effectiveness of psychiatric coercion, support offered, involvement of relatives, and level of knowledge of the laws authorizing psychiatric coercion). These results are presented in Table 3. The data demonstrate that the majority of participants perceive the support offered or received and the involvement of relatives during involuntary commitment and treatments to be low. The clinical effectiveness of involuntary commitment and treatments, as well as the level of knowledge of the associated laws, is viewed more favorably by most nurses (good) compared to patients (low).

**Table 1.** Sociodemographic Profile of Participants (Nurses).

Sociodemographic profile of participants (nurses, *n* = 10)	
Age	20–29 (*n* = 3)
	30–39 (*n* = 4)
	40–49 (*n* = 3)
Gender identity	Male (*n* = 3)
	Female (*n* = 7)
Level of education	College diploma (*n* = 2)
	University diploma, undergraduate level (*n* = 7)
	University diploma, graduate level (*n* = 1)
Years of experience in mental health	0–5 (*n* = 2)
	6–10 (*n* = 5)
	11–15 (*n* = 1)
	16–20 (*n* = 2)
Work environment	Emergency room (*n* = 2)
	Psychiatric unit (*n* = 6)
	Assertive community treatment team (*n* = 2)

**Table 2.** Sociodemographic Profile of Participants (Patients).

Sociodemographic profile of participants (patients, *n* = 11)	
Age	18–29 (*n* = 1)
	30–39 (*n* = 0)
	40–49 (*n* = 4)
	50–59 (*n* = 3)
	60–69 (*n* = 3)
Gender identity	Male (*n* = 5)
	Female (*n* = 5)
	Non-binary (*n* = 1)
Level of education	High school diploma (*n* = 2)
	College diploma (*n* = 5)
	University diploma (*n* = 4)
Marital status	Single (*n* = 9)
	Divorced (*n* = 2)
Occupation	Retired (*n* = 4)
	Unemployed (*n* = 3)
	Employed (*n* = 2)
	Disability benefits (*n* = 2)
Residential situation	Lives alone (*n* = 9)
	Joint tenancy (*n* = 2)
Services currently received	Institutional mental health services (*n* = 6)
	Community mental health services (*n* = 3)
	No services currently received (*n* = 5)
Types of coercion	Has experienced involuntary commitment (*n* = 8)
	Has experienced involuntary treatment (*n* = 4)
	Has experienced involuntary commitment and treatments (*n* = 3)

**Table 3.** Participants’ Perceptions of Psychiatric Coercion.

Perception of psychiatric coercion	Low	Good	Very good
Clinical effectiveness of psychiatric coercion	Patients = 10	Patients = 0	Patients = 1
	Nurses = 4	Nurses = 5	Nurses = 1
Professional support received or offered during involuntary hospitalizations or treatment	Patients = 10	Patients = 0	Patients = 1
	Nurses = 8	Nurses = 2	Nurses = 0
Family involvement	Patients = 8	Patients = 0	Patients = 3
	Nurses = 7	Nurses = 1	Nurses = 2
Perceived knowledge of laws authorizing psychiatric coercion	Patients = 7	Patients = 2	Patients = 2
	Nurses = 1	Nurses = 7	Nurses = 2

Finally, situational analysis revealed four conceptual categories, which will be detailed in the following sections. The first conceptual category, “Psychiatry as a Waiting Room,” represents the situation that emerged through the analysis of the data. The three other conceptual categories, “Nurses as Subordinates,” “Nothing Else but Medication,” and “Resisting Undignifying Care,” represent distinct dimensions that constitute this situation. All excerpts presented in this article are in French and have been freely translated by the authors.

### Psychiatry as a Waiting Room

Participants reported that services are characterized by a crisis-management logic. They also noted that no other forms of care are provided in relation to the elements that contributed to the crisis. As a result, psychiatric wards are seen as places where people are waiting to get better:

What I realized as a patient is that psychiatry is just a waiting room, hoping that the crisis will pass. They just observe, and when the crisis is over, they let you go without providing any care. (Glenn, patient)

These findings are corroborated by several nurses who participated in the study. They describe many situations where patients are abandoned to legal procedures, as if refusing hospitalization or treatment against their will was incompatible with any other form of support:

Often, people stay for a month or two, and we’re just waiting because we face a refusal. Some of my colleagues will simply say: you’re under [involuntary commitment]. That’s their intervention. (Fallon, nurse)

Some participants also reported seeking help at emergency departments voluntarily for various conditions, only to find themselves hospitalized against their will in psychiatry when they expressed hesitation or refused the services offered. This request for help thus transforms into an episode where the patient’s difficulties are amplified rather than alleviated by the care practices:

I spent the night in the ER. It was atrocious, really atrocious. [. . .] I was on a stretcher with a dozen people crammed next to each other. [. . .] It’s impossible to talk to anyone. It’s like an aquarium. There are no windows, but we are being observed by the staff. (Micah, patient)

Participants experienced that psychiatric services are characterized by frequent recourse to coercion. They also identified there appears to be no psychosocial support offered to patients to facilitate their recovery when hospitalizations and treatments are imposed, explaining why psychiatry is compared to a waiting room.

### Nurses as Subordinates

A key factor emerging from our analysis is the coercive role attributed to nursing staff. On one hand, professional hierarchies dictate that everything must “really go through the psychiatrist” (Lake, nurse). In this context, nurses are tasked with observing and reporting the patient’s condition to physicians so that they can “make an informed decision” (Blake, nurse), as well as “pacifying, calming down [feelings of anger] and administering medication” (Grey, nurse):

Either the doctor asks us to forcibly administer medication, or it’s a judge. Or a doctor decides to keep the person against their will, and we have to explain to them (patients) that it’s for their own good. So, we are somewhat caught between a rock and a hard place. (Quinn, nurse)

Unfortunately, it’s often the nurse who ends up constantly enforcing the rules. I feel like the bad cop, always saying: no, you can’t smoke, no, you can’t go outside, no, you can’t have your clothes. (Dane, nurse)

The patients participating in this study corroborated the subordinate role of nursing staff, whose coercive function limits their ability to support and assist those hospitalized or treated against their will. Some patients and nurses attributed this situation to the workload of the nursing staff, which is perceived as reducing the time available for patients in favor of managing the daily life of the units:

The nursing staff tortures us and forces us to take [medications] that we don’t want to take. They tie us up without valid reason, without justification. It’s torture, and people are scared. It’s not pleasant to be tied up. (River, patient)

How can they support us? [The nursing staff] is so overwhelmed. . . Nurses spend half their day handing out soap and opening the bathroom door. They do what they can, but they can’t help me or support me. Aside from giving me my pills, taking my blood pressure, and opening the bathroom door, there’s not much else they can do. (Glenn, patient)

For many participants of this study, the organization of work within mental health services appears to be a particularly significant factor in explaining the constraints on the professional autonomy of nursing staff. Participants identified that institutional expectations direct the daily activities of nurses toward technical, risk management, and security-oriented tasks rather than relational ones.

### Nothing Else but Medication

Psychiatry is described by several participants as a dead-end where nursing care is limited to pharmacotherapy: “They have to stay in the hospital, but we don’t have much to offer. [. . .] We’re short of nurses, so there’s not always someone to sit with them, and because we’re short-staffed, we have nothing to offer except medication” (Grey, nurse).

For many participants, involuntary commitment and treatments are accompanied by a lack of support. Moreover, a majority of patients confirmed the dominance of the biomedical approach at the expense of recovery-oriented practices. The analysis of case law also confirmed the biomedical orientation of court-ordered treatments:

In psychiatry, they don’t listen at all. They only have pills and injections. Those are the treatments. There’s nothing else. We’re bored to death, there’s nothing to do. Everything is centered around medication. There’s nothing to help you recover. (Chandler, patient)

The narratives collected in this study also challenge the interpersonal competencies of nursing staff. Patients felt over-responsible for their condition and behavior without any consideration for the underlying causes and sense-making of their distress:

When I started being hospitalized, [the staff] told me that I wasn’t behaving properly, that I wasn’t making the right choices in life or behavior. No one ever asked me why I was crying, what made me cry, or why I was sad. [. . .] They didn’t support me. . . I wasn’t supposed to feel the way I felt. (Harper, patient)

For many patients who took part in this study, the reliance on medication appears to be the sole response to their distress when they were hospitalized or treated against their will in psychiatry. The practices described by participants are also characterized by a lack of listening and support that would have helped them make sense of their experiences.

### Resisting Undignifying Care

Many patients associate psychiatry with practices more restrictive than imprisonment, preventing them from meeting their needs or maintaining their daily habits:

I lost all my rights. . . I wasn’t even allowed to go have coffee in the cafeteria when I was in [psychiatry]. I wasn’t allowed to have other clothes or go get them. Worse than a prisoner, because prisoners can go outside. I couldn’t. (Charlie, patient)

Several participants also reported having experienced trauma following one or more admissions to psychiatry. For them, the refusal of care seems to be more about protesting the current violence of psychiatry than refusing to receive help and support. For some, care practices end up amplifying and reactivating distress rather than alleviating it:

It’s like going to a doctor because you have a broken arm, and he breaks the other one: Yes, but I fixed this arm while breaking the other one. Ok, but I still have a broken arm. [. . .] If you’re admitted to psychiatry, [. . .] it’s because you’ve surely experienced trauma. [In psychiatry], they create more trauma, which is somewhat counterproductive. (Harper, patient)

However, nurses who participated in this study demonstrated professional autonomy and strategic actions aimed at preserving the dignity of patients, particularly when patients wished to contest legal procedures initiated by the psychiatric institution:

If they are admitted against their will, I often tell them how to [challenge the court’s decision]. I explain, I bring out the documents. . . if they can’t do it, I do it with them. [. . .] I just seem like someone causing trouble, but my colleagues are used to me now. It’s tolerated. (Roan, nurse)

Participants acknowledged that minimal support is offered to people hospitalized or treated against their will in psychiatry. This support is limited to providing general information about these measures rather than facilitating the exercise of human rights:

The staff gives you basic explanations about what’s going to happen, but that’s it. It’s very basic. They won’t provide you with tools to defend yourself. That’s not the treatment team’s mandate. (Cameron, patient)

Results suggest that the refusal of care is primarily attributed to the contestation of practices that undermine patients’ dignity and may even exacerbate their distress, rather than a refusal to receive support. However, practices of resistance were reported by participants in this study. Some nurses claim to take measures to better support and assist patients in understanding and, at times, contesting the legal measures imposed upon them.

## Discussion

The findings from this situational analysis reveal troubling conclusions regarding the quality of services provided to patients admitted or treated involuntarily in psychiatry. Our results suggest that the ethical issues surrounding involuntary commitment and treatments can be explained by how care is conceived. Care practices come to be limited to the application of coercive power delegated by the state (Jenkins et al., 2023), including the forced administration of medication. The distress potentially induced by involuntary commitment and treatments in patients comes to be ignored in favor of compliance with the legal procedures associated with these exceptional measures (Looi et al., 2014). Consequently, care practices appear to serve the interests of medical and judicial institutions (Adam et al., 2024; Barker & Buchanan-Barker, 2011) rather than benefiting the patients themselves (Crowe, 2022; Wand et al., 2022). The analogy comparing psychiatric services to a waiting room serves as a red flag, reiterating the detrimental effects of psychiatric coercion on patients, particularly when it is not accompanied by the necessary support to mitigate its negative effects (Aragonés-Calleja & Sánchez-Martínez, 2024). The experiences of patients emphasize a lack of assistance and support from nurses. Furthermore, our results highlight that a significant issue related to this problem is the loss of dignity experienced by patients (Plunkett & Kelly, 2021) in addition to psychiatric coercion itself.

The findings of our research suggest that nurses’ allegiance to the biomedical approach significantly reduces, rather than expands, their scope of action (Wand, 2024). Our findings align with those of several authors (Emerson & Pollner, 1976; Godin, 2000; McKeown, 2024), who suggest that mental health nurses are relegated to dirty work. They provide more precise documentation of the consequences of this dynamic on the quality of mental health care. The results of our situational analysis suggest a lack of recovery-oriented and trauma-informed care, not only because of a lack of resources but also because of the dominance of the biomedical authority and consequent constraints related to work organization, professional hierarchies, and institutional expectations. This situation hinders the deployment of person-centered practices by nurses, requiring their interventions to align with medical and judicial imperatives (Haslam & Harding, 2024; Wand et al., 2022) and thus contributing to the bullshitisation of nursing care (McKeown, 2024). This dynamic appears to contribute to the partial or complete absence of services addressing the needs of patients hospitalized and treated involuntarily, aside from pharmacotherapy. These findings echo numerous studies confirming the unpopularity of mental health nursing practice, characterized by attrition and stigma by association (Bujold et al., 2020; Njaka et al., 2023; Waddell et al., 2020).

Our results also suggest that some nurses exercise significant discretion when implementing involuntary commitment and treatments (Haslam & Harding, 2024; Lipsky, 2010). As this discretion stems from the delegation of public authority, its exercise can reinforce institutional logic (Haslam & Harding, 2024). However, our findings suggest that it may also serve as a form of silenced resistance (Crowe, 2022), which challenges the assertion that nurses comply with legal demands or medical authority without exercising their agency (Jenkins et al., 2023). In this context, such resistance is aimed at sharing necessary information to help patients better understand their situation, tailoring interventions to their needs, and providing support throughout the judicial process. Nevertheless, such interventions do not appear to characterize the experiences of the patients who participated in this study and remain marginal within the collected data. This situation may be explained by the complicity of nurses in the implementation of involuntary commitment and treatments, and by their internalization of the hegemonic discourse of medicine (Crowe, 2022). It may also be attributed to the disruptive potential of resistance practices to the daily routine and culture of teamwork, requiring nurses to take risks in their professional practice. These practices appear to induce a sense of professional deviance, which warrants further exploration.

Finally, the findings presented in this article shed new light on the phenomenon of refusing care and services in mental health. The results highlight certain causes related to the so-called refusal of psychiatric hospitalization or treatments. Warin’s (2016) work on the non-take-up of social policies suggests that the situation may be explained by more than just a refusal to seek support. These findings raise human dignity issues that compound the loss of freedom. Our results suggest that patients’ reluctance to use the services required by their mental health condition is associated with active resistance toward undignifying care. In this context, non-take-up could be explained by the absence of support, degrading practices, and a lack of therapeutic alternatives, rather than a refusal to receive help. Our study corroborates the findings of Silva et al. (2023) indicating that, when left on their own, patients describe their experience of hospitalization as a violation of their rights regardless of their legal status, characterized by a sense of disempowerment and vulnerability detrimental to their recovery. Our research also suggests that psychiatric coercion fosters a sense of objectification among patients (Adam et al., 2024; Sørås & Snipstad, 2021). This perception may lead patients to further resist proposed treatments to protect their identity and, in turn, objectify staff, thereby undermining the development of trusting relationships and hindering recovery (Sørås & Snipstad, 2021).

It could be argued that our research provides evidence supporting the integration of recovery-oriented and trauma-informed care principles during involuntary commitment and treatments. Similar to several authors (Adam et al., 2024; Barker & Buchanan-Barker, 2011; Crowe, 2022; Wand, 2024), we argue that integration is not sufficient and that an urgent paradigm shift is necessary in psychiatric nursing. We contend that the integration of recovery-oriented discourse into psychiatric care can create an illusion that “perpetuates the dominance of psychiatric discourse and position recovery principles as a peripheral value with limited impact on mental health services” (Crowe, 2022, p. 1546). The results presented in this article underscore the importance of psychiatric nursing adopting models of practice distinct from traditional psychiatric discourse. These models should acknowledge the trauma that precedes and results from a coercive episode, promote a better balance of power, foster trusting relationships, and actively support recovery through individualized care (McKay et al., 2021; Prytherch et al., 2021). Based on our results, key elements guiding psychiatric nursing practices include informing patients about their rights, empowering their participation if they wish to appeal the court’s decision, providing emotional support, and developing alternatives to medication-based interventions (Lakeman et al., 2023; Silva et al., 2023).

Our findings finally emphasize the need to avoid individualizing structural issues related to how psychiatric care is designed and delivered by psychiatric institutions. The sustainability of recovery-oriented, trauma-informed, and human-rights-based practices depends on adequate institutional orientations and support (Crowe, 2022; Wand et al., 2022). Psychiatric institutions must create enabling conditions for psychiatric nurses to engage effectively in such practices.

### Strengths and Limitations

To our knowledge, this is the first study in psychiatric nursing that questions the characteristics of interventions provided during episodes of involuntary commitment and treatments while examining care refusal through the lens of non-take-up of services. However, this research presents certain limitations. For ethical reasons associated with free and informed consent, patients were recruited through community mental health advocacy organizations rather than on psychiatric units. As a result, their experiences may not reflect those of other patients who were subjected to involuntary commitment and treatments. In addition, conducting observations within psychiatric units, as well as consulting internal documentation of psychiatric institutions—including procedures and task definitions—could have facilitated enhanced data triangulation and interpretation. Finally, a comparative perspective differentiating the experience of patients and nurses according to the type of measure used, involuntary commitment or treatments, would have helped to better qualify some of the results.

### Implications

The findings discussed in this article suggest several implications for research, social policies, and care practices. Regarding research, further studies are needed to explain what might counteract the subordination of nurses, the sense of deviance induced by practices informed by human rights, and the decision-making process in this context. As for social policies, our results suggest the relevance of integrating a plurality of therapeutic approaches to improve the adaptability of services and prevent non-take-up, particularly regarding recovery-oriented and trauma-informed care. Finally, the findings of our research argue for the development of new intervention strategies aimed at better assisting and supporting patients hospitalized and treated involuntarily in psychiatry.

## Conclusion

The nursing profession’s march toward recognition has historically been aligned with the biomedical paradigm, and mental health nursing is no exception (Pariseau-Legault & Paradis-Gagné, 2024). This choice of allegiance involves several sacrifices that may artificially constrain rather than support professional autonomy. The most important of these sacrifices is associated with the quality of services offered to patients. On this point, the status quo is no longer possible when we consider the lack of support, the absence of alternative therapeutic strategies, and the potentially traumatic consequences associated with involuntary commitment and treatments. Moreover, it is worth reiterating that these are exceptional measures of last resort which, although they are currently part of nurses’ work by being described as dirty work or even a necessary evil, do not imply any generalized suspension of human rights, including the right to dignity.

## Supplemental Material

sj-pdf-1-cnr-10.1177_10547738251321067 – Supplemental material for When Psychiatric Services Become a Waiting Room: Situational Analysis of Involuntary Commitment and Treatment as Experienced by Patients and Nurses Supplemental material, sj-pdf-1-cnr-10.1177_10547738251321067 for When Psychiatric Services Become a Waiting Room: Situational Analysis of Involuntary Commitment and Treatment as Experienced by Patients and Nurses by Pierre Pariseau-Legault, David Pelosse, Emmanuelle Bernheim, Marie-Hélène, Guillaume Ouellet, Lisandre Labrecque-Lebeau, Jean-Daniel Jacob and Dave Holmes in Clinical Nursing Research

sj-pdf-2-cnr-10.1177_10547738251321067 – Supplemental material for When Psychiatric Services Become a Waiting Room: Situational Analysis of Involuntary Commitment and Treatment as Experienced by Patients and Nurses Supplemental material, sj-pdf-2-cnr-10.1177_10547738251321067 for When Psychiatric Services Become a Waiting Room: Situational Analysis of Involuntary Commitment and Treatment as Experienced by Patients and Nurses by Pierre Pariseau-Legault, David Pelosse, Emmanuelle Bernheim, Marie-Hélène, Guillaume Ouellet, Lisandre Labrecque-Lebeau, Jean-Daniel Jacob and Dave Holmes in Clinical Nursing Research
