# Supplementary material for: When Psychiatric Services Become a Waiting Room: Situational Analysis of Involuntary Commitment and Treatment as Experienced by Patients and Nurses
Source: Clin Nurs Res. 2025 Mar 12;34(3-4):168–78. doi: 10.1177/10547738251321067 (PMC12053111; doi:10.1177/10547738251321067)
Supplement: sj-pdf-1-cnr-10.1177_10547738251321067 – Supplemental material for When Psychiatric Services Become a Waiting Room: Situational Analysis of Involuntary Commitment and Treatment as Experienced by Patients and Nurses [file sj-pdf-1-cnr-10.1177_10547738251321067.pdf]

Ordered Situational Map

Individual Human Elements / Actors	Nonhuman Elements Actors/Actants
Healthcare professionals (hospitalization units) –	Regional and geographical disparities
Healthcare professionals (community)	Organization of community services
Community workers	Organization of mental health services
Police officers	Hospital units
Managers	Housing (independent, supervised)
Family, friends and relatives	Courts
Lawyers of the healthcare institution	Professional hierarchies
Mental health advocates	Legal infrastructure related to psychiatric coercion
Judges	Psychiatric coercion as a clinical object
Principal researcher and research team	Psychiatric coercion as a legal object
**Collective Human Elements / Actors**	**Implicated/Silent Actors/Actants**
Human rights training program	Housing and socio-economic conditions
Healthcare institutions	Processes of discrimination and marginalization
Healthcare institution litigation	Accessibility of community organizations
Community organizations	Professional corporatism
Police forces	Professional ethics
Professional orders and associations	Culture of practice in psychiatry
Work teams	Best practices discourse
Advocacy organizations	Right-based discourse
Coroner's office	Media discourse
**Discursive Construction of Individual and/or Collective Human Actors**	**Discursive Construction of Nonhuman Actants**
Legal experts	Lack of access to mental health services
Managerial experts	Exclusion of non-institutional practices
Psychiatric experts	Biomedical psychiatry as a problematic model of care
Coercive agents (street-level bureaucrats)	Biomedical psychiatry as a unique model of care
Supportive agents	Positive consequences of psychiatric coercion
Disruptive agents	Negative consequences of psychiatric coercion
Users in need of protection	Psychiatric coercion as a last resort intervention
Friends, family and relatives as external consultants	Psychiatric coercion as a necessary evil
	Psychiatric coercion as a dominant practice
	Trivialization of social exclusion dynamics
**Political/Economic Elements**	**Sociocultural/Symbolic Elements**
Social and economic precariousness	Poverty
Issues with access to mental health services	Social isolation and exclusion
Single, medication-centered care model	Discrimination
Intersectoral collaboration	Marginalization
Interministerial action strategy on mental health	Collective relationship to mental health and illness
Lack of recognition for mental health advocacy practices	Judicialization of social issues
Lack of resources	Technical role assigned to healthcare staff
Moral division of labor	Courts' decorum and procedures
Staff turnover	Self-presentation (hearings)
Risk management	
Public inquiries related to evitable deaths	
Case law	
Framework for implementing psychiatric coercion	
Discourse focused on public safety	
Discourse focused on risk management	
Ambiguous and indeterminate nature of dangerousness and psychiatric risk	

Temporal Elements	Spatial Elements
First Quebec laws on asylums and involuntary commitment (1851)<br>Mental Patients Protection Act (1972-1997)<br>Act respecting the protection of persons whose mental state presents a danger to themselves or to others (1997 – Present)<br>Entry into force - Civil Code of Quebec (1994)<br>Economic, cultural, and social rights (1976)<br>Public inquiry report on the challenges of applying psychiatric law in Quebec, Canada (2011)<br>Case law related to the challenges of applying psychiatric law in Quebec, Canada<br>Reference framework for the application of the Act respecting the protection of persons whose mental state presents a danger to themselves or to others (2018)<br>Evolution of the State's social mission regarding mental health<br>Numerous reforms of health and social services	Spatial and temporal characteristics of hospital units<br>Judicial care authorizations create an open control space in the community<br>Courts and court hearings<br>Court hearings by videoconference (COVID-19)<br>Relational spaces brought into play by psychiatric coercion
**Major Issues/Debates (Usually Contested)**	**Related discourses (Historial, Narrative, and/or Visual)**
Difference between rights support and rights defense practices<br>Disciplinary conflicts regarding rights as objects of appropriation, interpretation and intervention<br>Relative invisibility of nursing staff<br>Absence of a human rights-based approach in psychiatry<br>Coercion creates a lawless zone (state of exception)<br>Emphasis on meeting procedural demands<br>Exclusion of friends, family and relatives	Vulnerability<br>Risk (self, others)<br>Danger (self, others)<br>Mental health and illness<br>Legal expertise<br>Medical expertise<br>Human rights
**Other Kinds of Elements**	
Professional autonomy<br>Independent advocacy<br>Continuity/rupture of institutional and community practices<br>Dual role of nursing staff<br>Situations of non-take-up of services induced by coercion	

Adapted from: Clarke, A. (2005). *Situational Analysis.* SAGE Publications.

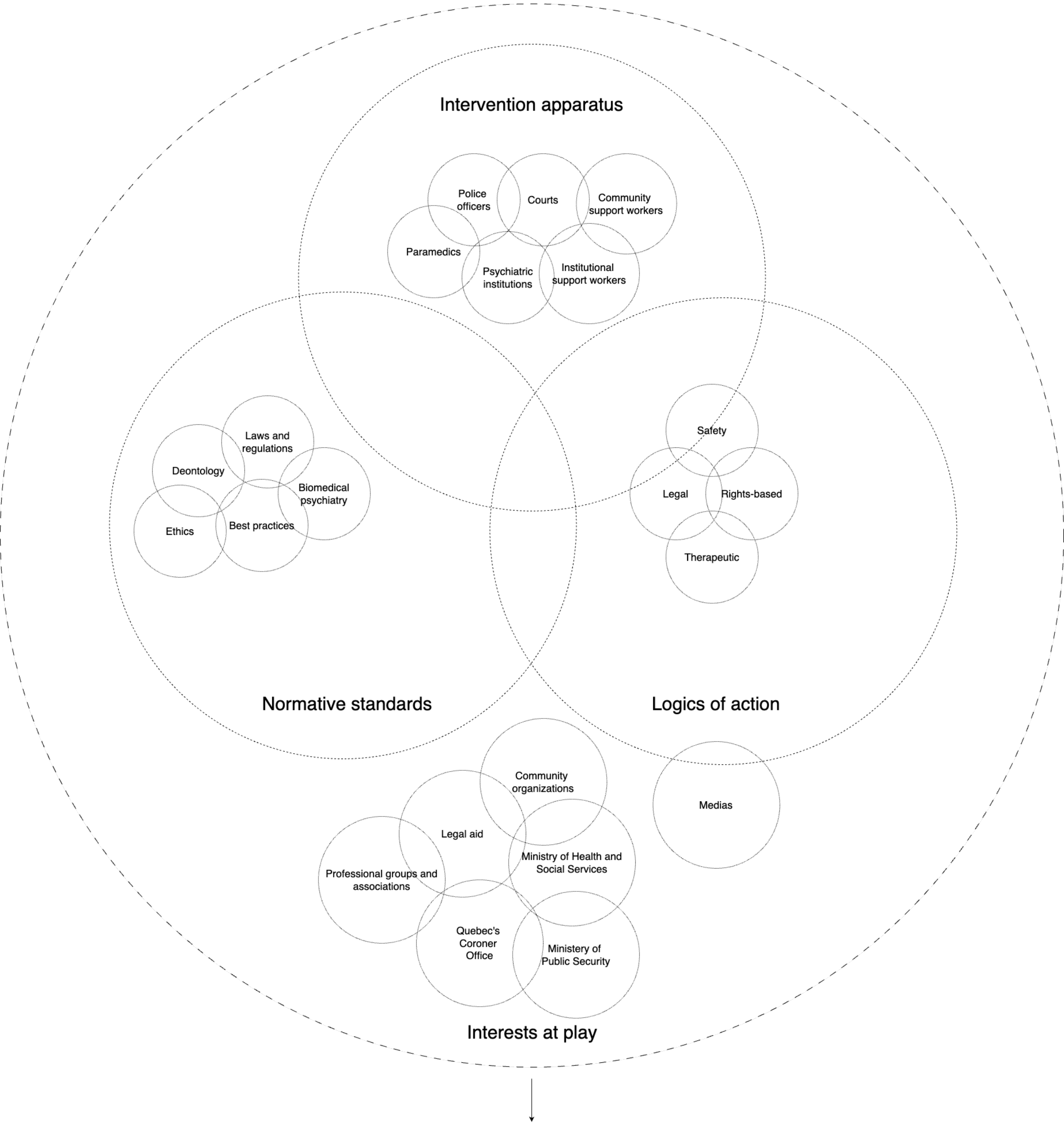

Benevolent rhetoric

Social worlds/arenas map

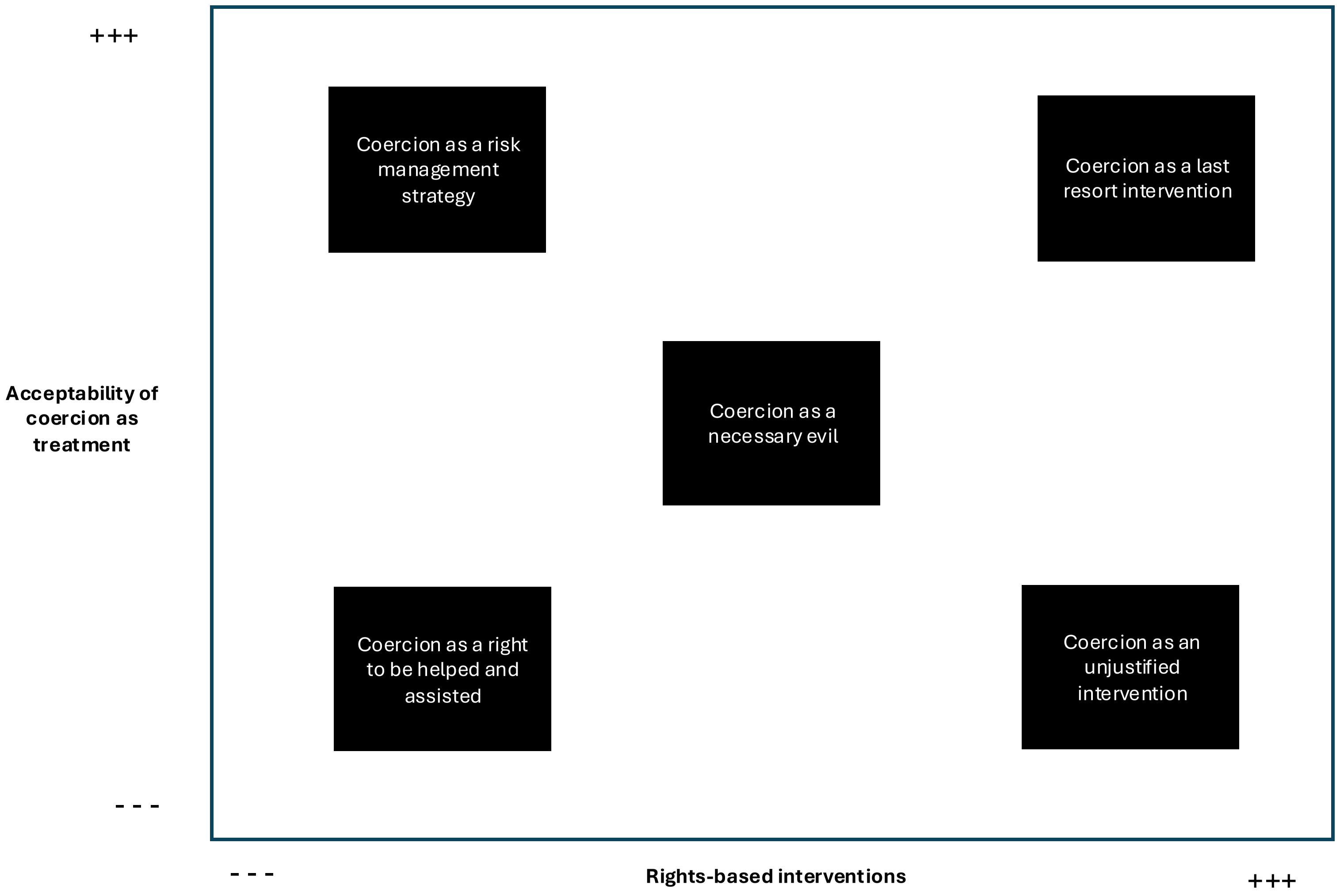

Positional map
Implementation of involuntary commitment and treatment
