# Supplementary material for: When Psychiatric Services Become a Waiting Room: Situational Analysis of Involuntary Commitment and Treatment as Experienced by Patients and Nurses
Source: Clin Nurs Res. 2025 Mar 12;34(3-4):168–78. doi: 10.1177/10547738251321067 (PMC12053111; doi:10.1177/10547738251321067)
Supplement: sj-pdf-2-cnr-10.1177_10547738251321067 – Supplemental material for When Psychiatric Services Become a Waiting Room: Situational Analysis of Involuntary Commitment and Treatment as Experienced by Patients and Nurses [file sj-pdf-2-cnr-10.1177_10547738251321067.pdf]

Semi-structured interview questionnaire – Patients

General Experience
1. Tell me what happened when you were escorted, admitted, hospitalized or treated without your consent?<br>2. Why were you required to undergo escort, admission, hospitalization or treatment?<br>3. What was your experience like?<br>4. What difficulties did you encounter during your escort, admission, hospitalization or forced treatment?
Intervention practices during involuntary commitment and treatment
5. When the escort, admission, hospitalization and treatment were imposed, what care or services did you receive from support workers?<br>6. What could support workers do to better assist people going through the same thing as you?<br>7. During your escort, admission, hospitalization or forced treatment, who were the people you trusted to help you?<br>8. How has your experience of escort, admission, hospitalization or forced treatment affected the relationship you have with your support workers and psychiatric services in general?
Support for Exercising Rights during Involuntary Commitment and Treatment
9. Based on your experience, what impact do escorts, admissions, hospitalization or involuntary treatment have on your rights?<br>10. How did you defend your rights or receive support in exercising your rights during your experience of escort, admission, hospitalization or involuntary treatment?<br>11. How did you find out about your rights when the escort, admission, hospitalization or treatment was imposed?<br>12. If you disagreed with being hospitalized or receiving psychiatric treatment, how did you challenge this decision?<br>13. How do you feel about exercising your rights following your experience?<br>14. Based on your experience, how could support in exercising your rights be improved?
Mobilization of Support Systems
15. During your experience of escort, admission, hospitalization or forced treatment, what support did you receive from your family, friends or relatives?<br>16. How has your experience of escort, admission, hospitalization or forced treatment affected the relationship you have with your family, friends or relatives?
Concluding Questions
17. Is there anything you would like to discuss that you think is relevant but that we haven't yet talked about?<br>18. What would you like to say to people who, like you, have been subjected to psychiatric escort/admission, hospitalization or treatment without their consent?

Semi-structured interview questionnaire – Support workers

Intervention Practices
1. In what context do you provide support to individuals who have been escorted, admitted, hospitalized, or treated involuntarily in mental health settings?<br>2. Why and for what purpose do you resort to escort, admission, hospitalization, or involuntary treatment in mental health care?<br>3. Describe your role with the person during escort, forced admission, forced hospitalization, or when treatments are imposed or likely to be imposed.
Personal Experience
4. How do you experience such situations?<br>5. How do you personally and professionally cope with psychiatric coercion?<br>6. What do you take away from these experiences?
Support for Exercising Rights
7. What support do you provide to individuals who are escorted, admitted, hospitalized, or treated without their consent?<br>8. How do you support the exercise of the individual's rights when escort, admission, hospitalization, or treatment is imposed?<br>9. What is the impact of supporting the exercise of rights in coercive contexts on the individual's well-being and care trajectory?<br>10. How are connections established and maintained between the healthcare and judicial systems when an escort, admission, hospitalization, or treatment is imposed on the individual?
Mobilization of Support Systems
11. What is the influence of coercion on the therapeutic alliance or relationships between the individual and their surroundings?<br>12. Describe your role with the family and close associates of the individual during forced hospitalization or when treatments are imposed.
Concluding Question
13. So far, how have these experiences with coercion in mental health care changed, or not changed, your intervention approach?
